# Supplementary figures and images for: Clinical characteristics and disease course of splanchnic vein thrombosis in gastrointestinal cancers: A prospective cohort study
Source: PLoS One. 2022 Jan 18;17(1):e0261671. doi: 10.1371/journal.pone.0261671 (PMC8765650; doi:10.1371/journal.pone.0261671)

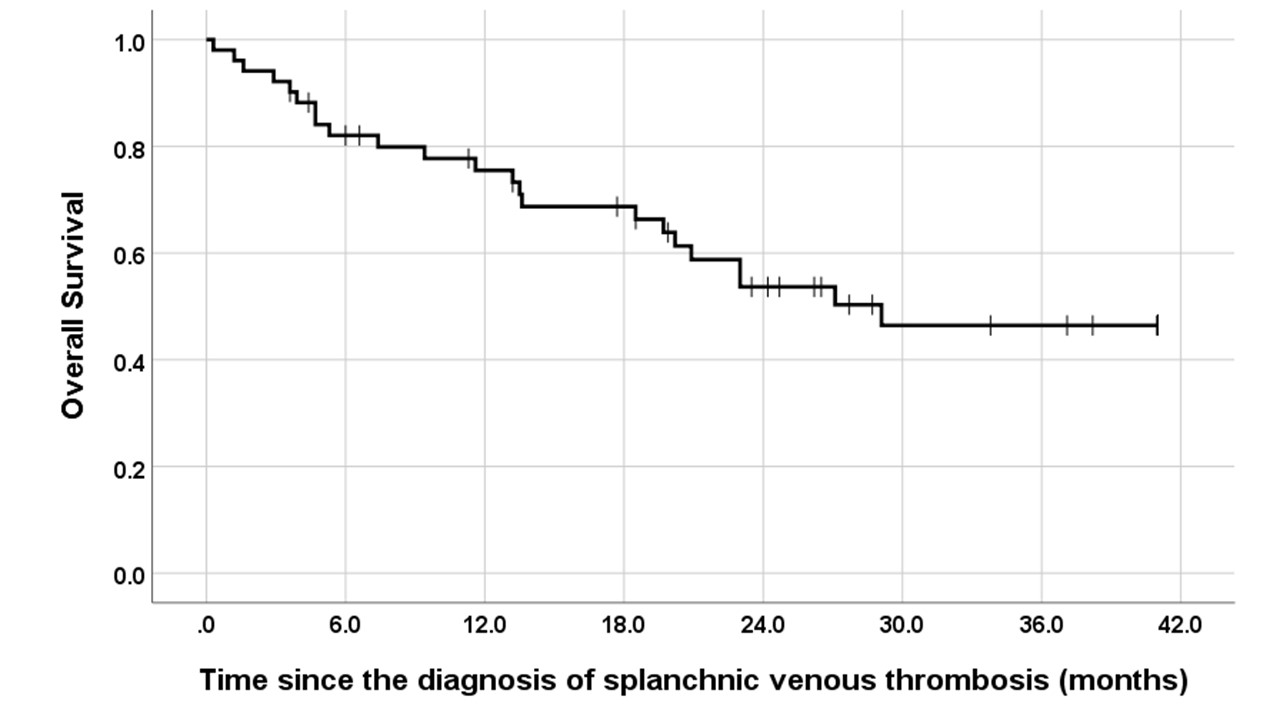

Supplement: S1 Fig — (JPG) [file pone.0261671.s001.JPG]

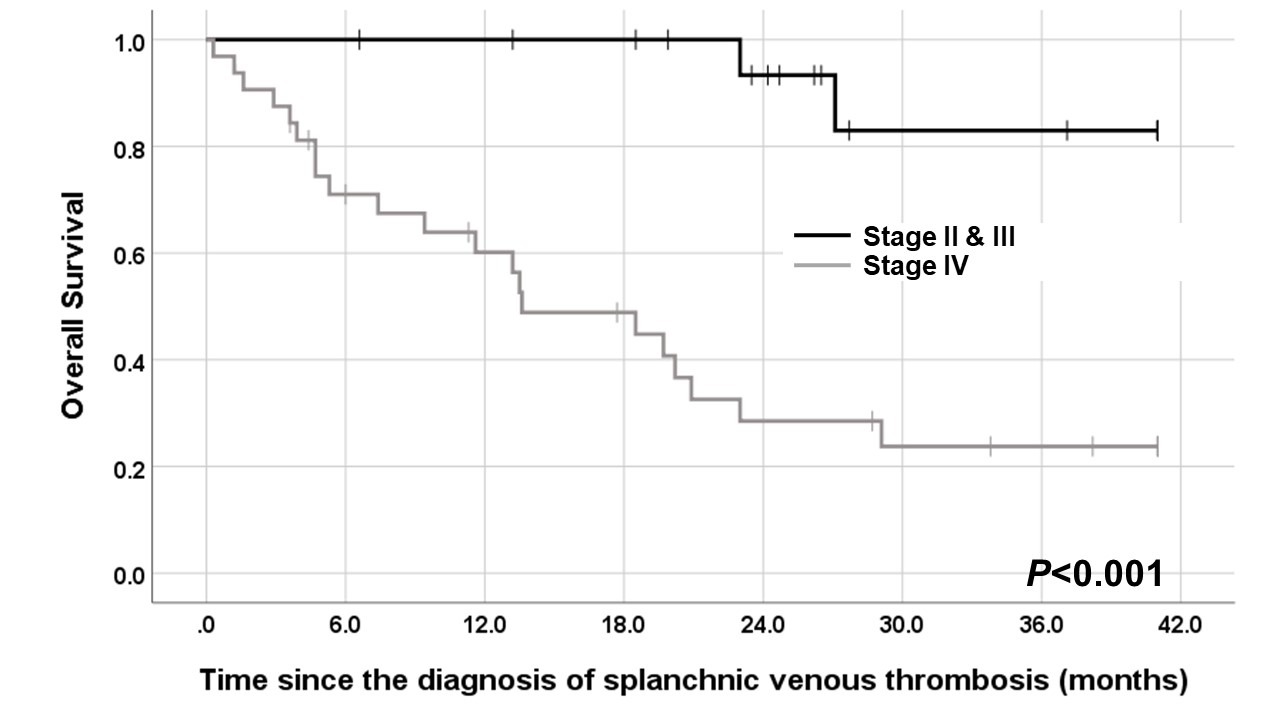

Supplement: S2 Fig — (JPG) [file pone.0261671.s002.JPG]
