# Supplementary material for: Clinical characteristics and disease course of splanchnic vein thrombosis in gastrointestinal cancers: A prospective cohort study
Source: PLoS One. 2022 Jan 18;17(1):e0261671. doi: 10.1371/journal.pone.0261671 (PMC8765650; doi:10.1371/journal.pone.0261671)
Supplement: S1 Table — (DOCX) [file pone.0261671.s003.docx]

**S1 Table.** Univariable and multivariable analyses on prognostic factors in patients with splanchnic vein thrombosis

|  | N | Median (months) | 3-yr OS rate | P |  | HR | 95% CI | P |
| --- | --- | --- | --- | --- | --- | --- | --- | --- |
| **Sex** |  |  |  | 0.920 |  |  |  |  |
| Male | 39 | 27.1 | 43.0% |  |  | - | - | - |
| Female | 12 | NR | 56.3% |  |  | - | - | - |
| **Age** |  |  |  | 0.011 |  |  |  |  |
| < 70 years | 41 | NR | 56.6% |  |  | 1.00 | - | - |
| ≥ 70 years | 10 | 13.2 | 0.0% |  |  | 2.02 | 0.80-5.13 | 0.139 |
| **ECOG performance status** |  |  |  | <0.001 |  |  |  | <0.001 |
| 0 | 15 | 29.1 | 49.2% |  |  | 1.00 | - | - |
| 1 | 30 | NR | 54.0% |  |  | 0.75 | 0.27-2.08 | 0.58 |
| ≥ 2 | 6 | 1.6 | 0.0% |  |  | 28.15 | 3.94-201.07 | 0.001 |
| **Primary tumor** |  |  |  | 0.231 |  |  |  |  |
| Gastric cancer | 25 | 20.2 | 42.1% |  |  | - | - | - |
| Colorectal cancer | 24 | 29.1 | 44.6% |  |  | - | - | - |
| Others | 2 | NR | 100.0% |  |  | - | - | - |
| **Tumor pathology** |  |  |  | 0.189 |  |  |  |  |
| WDAC/MDAC | 30 | NR | 57.1% |  |  | - | - | - |
| PDAC | 18 | 20.9 | 33.9% |  |  | - | - | - |
| Others | 3 | 20.2 | 33.3% |  |  | - | - | - |
| **Stage** |  |  |  | <0.001 |  |  |  |  |
| II/III | 19 | NR | 83.0% |  |  | 1.00 | - | - |
| IV | 32 | 13.6 | 23.7% |  |  | 7.67 | 1.64-35.97 | 0.010 |
| **Location of SpVT** |  |  |  | 0.979 |  |  |  |  |
| Portal vein | 34 | NR | 51.8% |  |  | - | - | - |
| Mesenteric vein (Superior or inferior) | 10 | 27.1 | 45.7% |  |  | - | - | - |
| Others | 3 | 29.1 | 33.3% |  |  | - | - | - |
| Multiple sites | 4 | 23.0 | 50.0% |  |  | - | - | - |
| **SpVT-related symptoms** |  |  |  | 0.003 |  |  |  |  |
| Absent | 46 | 29.1 | 50.3% |  |  | 1.00 | - | - |
| Present | 5 | 5.3 | 20.0% |  |  | 1.38 | 0.36-5.40 | 0.636 |
| **Clinical situation at the diagnosis of SpVT** |  |  |  | <0.001 |  |  |  |  |
| After surgery | 14 | NR | 78.8% |  |  | - | - | - |
| Initial diagnosis of cancer or tumor recurrence (after curative therapy) | 12 | 18.5 | 22.2% |  |  | - | - | - |
| During chemotherapy (without tumor progression) | 11 | NR | 87.5% |  |  | - | - | - |
| During chemotherapy (with tumor progression) | 10 | 5.3 | 15.2% |  |  | - | - | - |
| Terminal phase (no more chemotherapy) | 4 | 1.2 | 0.0% |  |  | - | - | - |
| **Albumin level (Serum)** |  |  |  | 0.180 |  |  |  |  |
| ≥ 3.0g/dL | 41 | 29.1 | 46.0% |  |  | - | - | - |
| < 3.0g/dL | 10 | 7.4 | 40.0% |  |  | - | - | - |
| **Hemoglobin level (Plasma)** |  |  |  | 0.052 |  |  |  |  |
| ≥ 10.0g/dL | 34 | NR | 53.0% |  |  | - | - | - |
| < 10.0g/dL | 17 | 18.5 | 33.1% |  |  | - | - | - |
| **White blood cell count level (Plasma)** |  |  |  | 0.395 |  |  |  |  |
| ≥ 4000/μL | 40 | NR | 55.4% |  |  | - | - | - |
| < 4000/μL | 11 | 23.0 | 15.7% |  |  | - | - | - |
| **Platelet count level (Plasma)** |  |  |  | 0.531 |  |  |  |  |
| ≥ 13,000/μL | 42 | 29.1 | 48.6% |  |  | - | - | - |
| < 13,000/μL | 9 | 23.0 | 40.0% |  |  | - | - | - |
| Abbreviations: SpVT, splanchnic vein thrombosis; ECOG, Eastern Cooperative Oncology group; WDAC, well differentiated adenocarcinoma; MDAC, moderately differentiated adenocarcinoma; PDAC, poorly differentiated adenocarcinoma. | | | | | | | | |
