# Supplementary material for: Clinical characteristics and disease course of splanchnic vein thrombosis in gastrointestinal cancers: A prospective cohort study
Source: PLoS One. 2022 Jan 18;17(1):e0261671. doi: 10.1371/journal.pone.0261671 (PMC8765650; doi:10.1371/journal.pone.0261671)
Supplement: S2 Table — (DOCX) [file pone.0261671.s004.docx]

**S2 Table.** Univariable and multivariable analyses on prognostic factors in patients with splanchnic vein thrombosis (repeated survival analysis with a short time of follow-up, 12 months)

|  | N | 12-month OS rate | P |  | HR | 95% CI | P |
| --- | --- | --- | --- | --- | --- | --- | --- |
| **Sex** |  |  | 0.364 |  |  |  |  |
| Male | 39 | 78.6% |  |  | - | - | - |
| Female | 12 | 65.6% |  |  | - | - | - |
| **Age** |  |  | 0.189 |  |  |  |  |
| < 70 years | 41 | 79.6% |  |  | - | - | - |
| ≥ 70 years | 10 | 60.0% |  |  | - | - | - |
| **ECOG performance status** |  |  | <0.001 |  |  |  | 0.010 |
| 0 | 15 | 86.2% |  |  | 1.00 | - | - |
| 1 | 30 | 85.7% |  |  | 0.78 | 0.12-5.08 | 0.790 |
| ≥ 2 | 6 | 0.0% |  |  | 32.19 | 1.78-583.83 | 0.019 |
| **Primary tumor** |  |  | 0.099 |  |  |  |  |
| Gastric cancer | 25 | 62.2% |  |  | - | - | - |
| Colorectal cancer | 24 | 86.9% |  |  | - | - | - |
| Others | 2 | 100.0% |  |  | - | - | - |
| **Tumor pathology** |  |  | 0.341 |  |  |  |  |
| WDAC/MDAC | 30 | 82.3% |  |  | - | - | - |
| PDAC | 18 | 64.2% |  |  | - | - | - |
| Others | 3 | 66.7% |  |  | - | - | - |
| **Stage^a^** |  |  | 0.002 |  |  |  |  |
| II/III | 19 | 100.0% |  |  | - | - | - |
| IV | 32 | 71.0% |  |  | - | - | - |
| **Location of SpVT** |  |  | 0.680 |  |  |  |  |
| Portal vein | 34 | 75.6% |  |  | - | - | - |
| Mesenteric vein (Superior or inferior) | 10 | 90.0% |  |  | - | - | - |
| Others | 3 | 66.7% |  |  | - | - | - |
| Multiple sites | 4 | 75.0% |  |  | - | - | - |
| **SpVT-related symptoms** |  |  | 0.026 |  |  |  |  |
| Absent | 46 | 82.0% |  |  | 1.00 | - | - |
| Present | 5 | 40.0% |  |  | 1.59 | 0.18-13.77 | 0.675 |
| **Clinical situation at the diagnosis of SpVT** |  |  | <0.001 |  |  |  | 0.023 |
| After surgery^b^; initial diagnosis of cancer or tumor recurrence (after curative therapy)^c^; during chemotherapy (without tumor progression)^d^ | 37 | 91.5% |  |  | 1.00 | - | - |
|  |  |  |  |  |  |  |  |
|  |  |  |  |  |  |  |  |
| During chemotherapy (with tumor progression) | 10 | 30.5% |  |  | 8.72 | 1.48-51.37 | 0.017 |
| Terminal phase (no more chemotherapy) | 4 | 25.0% |  |  | 16.32 | 1.64-163.01 | 0.017 |
| **Albumin level (Serum)** |  |  | 0.001 |  |  |  |  |
| ≥ 3.0g/dL | 41 | 84.5% |  |  | 1.00 | - | - |
| < 3.0g/dL | 10 | 40.0% |  |  | 13.50 | 2.38-76.63 | 0.003 |
| **Hemoglobin level (Plasma)** |  |  | 0.044 |  |  |  |  |
| ≥ 10.0g/dL | 34 | 84.4% |  |  | 1.00 | - | - |
| < 10.0g/dL | 17 | 58.8% |  |  | 0.46 | 0.084-2.534 | 0.374 |
| **White blood cell count level (Plasma)** |  |  | 0.213 |  |  |  |  |
| ≥ 4000/μL | 40 | 71.0% |  |  | - | - | - |
| < 4000/μL | 11 | 90.9% |  |  | - | - | - |
| **Platelet count level (Plasma)** |  |  | 0.963 |  |  |  |  |
| ≥ 13,000/μL | 42 | 74.8% |  |  | - | - | - |
| < 13,000/μL | 9 | 77.8% |  |  | - | - | - |
| Abbreviations: SpVT, splanchnic vein thrombosis; ECOG, Eastern Cooperative Oncology group; WDAC, well differentiated adenocarcinoma; MDAC, moderately differentiated adenocarcinoma; PDAC, poorly differentiated adenocarcinoma.  ^a^In the case of ‘stage’, there was a high correlation with ‘clinical situation at the diagnosis of SpVT’, so multicollinearity was concerned. Additionally, in stage II/III, there was no death event within 12 months. Therefore, ‘stage’ was not included in the multivariable analysis.  ^b, c, d^These 3 items were combined into one category because of the small death events in each situation. | | | | | | | |
