# Supplementary material for: Clinical characteristics and disease course of splanchnic vein thrombosis in gastrointestinal cancers: A prospective cohort study
Source: PLoS One. 2022 Jan 18;17(1):e0261671. doi: 10.1371/journal.pone.0261671 (PMC8765650; doi:10.1371/journal.pone.0261671)
Supplement: S1 Appendix — (DOCX) [file pone.0261671.s005.docx]

**S1 Appendix.** Post hoc power analysis

Since we planned to enroll as many SpVT patients as possible during the study period, sample size and power calculations were not performed at the start of this study. In this page, we summarized the results of post hoc analysis by setting the hypothesis as follows. PASS 2021, v21.0.2 was used for this calculation.

Null Hypothesis (H_0_): P_0_ of SpVT patients will need anticoagulant therapy.

Alternative hypothesis (H_1_): P_1_ of SpVT patients will need anticoagulant therapy.

Our study: 9 out of 51 patients (9/51, P_1_ = 0.176) received anticoagulant treatment.

**Numeric Results for Testing One Proportion using the Z-Test with S(P_0_) and Continuity Correction.** **─────────────────────────────────────────────────────**

Alternative Hypothesis: Two-Sided (H0: P = P_0_ vs. H_1_: P ≠ P0)

**Proportion Proportion**

**Given H_0_ Given H_1_ Difference Reject H_0_**

**Power* n P_0_ P_1_ P_1_-P_0_ Alpha If |Z| >**

0.15282 51 0.25 0.176 -0.074 0.05 1.96

0.41410 51 0.30 0.176 -0.124 0.05 1.96

0.73379 51 0.35 0.176 -0.174 0.05 1.96

0.93260 51 0.40 0.176 -0.224 0.05 1.96

0.99167 51 0.45 0.176 -0.274 0.05 1.96

0.99955 51 0.50 0.176 -0.324 0.05 1.96

* Power was computed using the normal approximation method.

**Summary Statements**

**─────────────────────────────────────────────────────**

(1) A sample size of 51 achieves 73.379% power to detect a difference (P_1_-P_0_) of -0.174 using a two-sided Z-test that uses S(P_0_) to estimate the standard deviation and has a continuity correction with a significance level (alpha) of 0.05. These results assume a population proportion of 0.35 under the null hypothesis (P_0_), which means that about one-third of patients will require anticoagulation.

(2) A sample size of 51 achieves 99.955% power to detect a difference (P_1_-P_0_) of -0.324 using a two-sided Z-test that uses S(P_0_) to estimate the standard deviation and has a continuity correction with a significance level (alpha) of 0.05. These results assume a population proportion of 0.50 under the null hypothesis (P_0_), which means that about half of patients will require anticoagulation.

**Chart Section ─────────────────────────────────────────────────────**
