# Supplementary material for: Clinical characteristics and disease course of splanchnic vein thrombosis in gastrointestinal cancers: A prospective cohort study
Source: PLoS One. 2022 Jan 18;17(1):e0261671. doi: 10.1371/journal.pone.0261671 (PMC8765650; doi:10.1371/journal.pone.0261671)
Supplement: Attachment — Submitted filename: Response to Reviewers.docx [file pone.0261671.s007.docx]

-August, 2021

**Editor-in-Chief: Dr. Emily Chenette; *PLOS ONE***

**Dear Dr. Chenette,**

Thank you very much for your insightful review of our manuscript. All the comments were very helpful to improve the quality of this report, and we did our best to address your detailed comments and suggestions. Adhering to the respectful recommendations suggested by the Editor and the reviewers, we have revised our manuscript. Point-by-point revisions are outlined in detail below.

All of the authors have read and agreed with the revised manuscript. Again, thank you very much for considering our work for publication in your journal. This research was partially funded by the Seoul National University Bundang Hospital Research Fund (Number: 02-2017-036). There was no additional external funding received for this study.

Along with my coauthors, I would like to ask you to consider the attached manuscript for publication in ***'PLOS ONE***' as an original article.

Best regards,

Keun-Wook Lee, M.D., Ph.D.

Professor, Department of Internal Medicine, Seoul National University Bundang Hospital, Seoul National University College of Medicine

82 Gumi-ro 173 Beon-gil, Bundang-gu, Seongnam 13620, Republic of Korea

Phone: +82-31-787-7039; E-mail: hmodoctor@snu.ac.kr

PONE-D-21-10010

Clinical characteristics and disease course of splanchnic vein thrombosis in gastrointestinal cancers: A prospective cohort study

PLOS ONE

**Academic editor:** However, the reviewers raise some issues that needs to be addressed. In addition to these, please provide any information, in available, on presence of other thrombophilia factors.

***=> Reply)*** *Thank you for the important comment. We added one sentence (‘^c^No patient had thrombophilic disorders’) to the footnote of the table (Page 7; Table 1).*

**Journal Requirements:**

#1) Please provide a sample size and power calculation in the Methods, or discuss the reasons for not performing one before study initiation

***=> Reply #1)*** *This was a simple observational study. So, the formal sample size calculation was not done. The following sentence was added in the revised manuscript.*

*; Since it was planned to enroll as many SpVT patients as possible during the study period, sample size and power calculations were not performed at the start of this study (Page 4, Line 75-77).*

#2) Thank you for stating in your Funding Statement: "This research was partially funded by the Seoul National University Bundang Hospital Research Fund (Number: 02-2017-036). The funders had no role in study design, data collection and analysis, decision to publish, or preparation of the manuscript."

Please provide an amended statement that declares *all* the funding or sources of support (whether external or internal to your organization) received during this study, as detailed online in our guide for authors at http://journals.plos.org/plosone/s/submit-now. Please also include the statement “There was no additional external funding received for this study.” in your updated Funding Statement. Please include your amended Funding Statement within your cover letter. We will change the online submission form on your behalf.

***=> Reply #2)*** *In acknowledgements, the funding source was more clarified per the request. Also, Acknowledgments were added to Medical Research Collaborating Center at Seoul National University Bundang Hospital for helping with statistical analysis in the revision of this manuscript as below.*

*; This research was partially funded by the Seoul National University Bundang Hospital Research Fund (Number: 02-2017-036). There was no additional external funding received for this study. The authors thank all patients who participated in this study. We are grateful to Medical Research Collaborating Center (MRCC) at Seoul National University Bundang Hospital for the assistance in the revision of this manuscript.*

**Reviewer #1**:

The study involves a prospective analysis of a small cohort of subjects with splanchnic vein thrombosis, mainly in gastric and colorectal cancers. Although limited by its size, it is technically sound with appropriate statistical analysis and presented in an easily comprehensible manner. I would request the authors to clarify a couple things.

*=> Thank you for the constructive and valuable comments and suggestions. We revised our manuscript following your suggestions. Please refer to the manuscript file with track changes.*

**#1-1)** Line 148, Page 9- What does 'aggravation' of SpVT mean? Does it mean extension of SpVT or development of new SpVT related symptoms or both?

***=> Reply #1-1)*** *Thank you for the important comment. The expression 'aggravation of SpVT' appears first in Table 2. Reflecting the reviewer's comment, we have added the additional comments in Table 2 as shown below. This was also reflected in the Page 10 (Line 159-160).*

***Changes in the text #1-1)***

***-*** *^k^The SpVT extended from the existing location in 2 patients with gastric cancer. In 3 patients (one with gastric cancer and two with colorectal cancer), development of new SpVT was observed. (Page 9; Table 2)*

*- In five other patients (9.8%), anticoagulation therapy was initiated at the time of SpVT aggravation (extension of pre-existing SpVT or development of new SpVT) (Page 10, Line 159-160).*

**#1-2)** Line 146, Page 9- Please clarify what lead to the 4 patients being treated with anticoagulation upon initial diagnosis? Were they all symptomatic or had concomitant DVT/PE or any other factors?

***=> Reply #1-2)*** *We appreciate your important comments. Reflecting the reviewer’s comment, we have revised the manuscript as shown below.*

***Changes in the text #1-2)***

*Four patients (7.8%) received anticoagulation treatment at the time of initial SpVT diagnosis: in 2 patients, SpVT was incidentally detected after surgery and anticoagulant was performed considering the possibility of worsening in the postop period; in the other two cases, SpVT was identified with tumor progression and anticoagulation was conducted for symptom management (in the first patient, ascites accompanying portal vein thrombosis was observed, and in the other case, DVT was detected simultaneously with SpVT). (Page 10, Line 153-158)*

**#1-3)** Did any patients in the study suffer from SpVT related complications such as mesenteric ischemia or portal hypertension?

***=> Reply #1-3)*** *Thank you for the important comment. As noted in Table 2, at the time of initial diagnosis of SpVT, 5 patients had abdominal symptoms, and two of them had portal vein thrombus and ascites. In addition, during the follow-up period, 2 patients developed new SpVT-related symptoms and one of these two patients had portal vein thrombosis and ascites. These three patients may have developed ascites due to portal hypertension. However, as described in discussion section, in cases with abdominal discomfort or ascites, distinguishing whether these symptoms were caused by tumors (i.e., peritoneal seeding) or portal hypertension due to portal vein thrombosis was difficult. Additional descriptions for this were not added to the revised manuscript.*

*In our patient cohort, we observed one case with mesenteric ischemia. One patient (M/58) with stage IV gastric cancer developed asymptomatic portal vein thrombosis during palliative chemotherapy. However, during the chemotherapy, severe abdominal pain and ileus developed and new superior mesenteric vein thrombosis was detected. In this case, mesenteric ischemia was strongly suspected and improved after use of dalteparin. This description was added to the footnote of Table 2 with the superscript ‘g’ (Page 9; Table 2).*

**Reviewer #2**:

*=> Thank you for the constructive and valuable comments and suggestions. We revised our manuscript following your suggestions. Please refer to the manuscript file with track changes.*

Major comments:

In the "Materials and methods" section please specify:

**#2-1)**
- if patients is consecutively enrolled
- the outcome of interest. Authors reported in the Results mortality rate, recurrent venous thrombotic events, and the rate of vessel recanalization without any information in the Material and methods section. Furthermore, authors should report (if available) major and clinically relevant non-major bleeding occurred during follow-up.

***=> Reply #2-1)*** *We appreciate your constructive suggestion. Reflecting the reviewer’s comments, we have added the additional comments to "Materials and methods" section as shown below.*

***Changes in the text #2-1)*** *- The patients were consecutively enrolled between June 2017 and July 2020. (Page 4, Line 74-75)
- The outcomes of interest are described as below;*

*; The outcomes of interest in this observational study were clinical characteristics and course of SpVT, and risk factors influencing survival in patients with SpVT (Page 5, Line 93-94).*

*; OS was defined as the time period between the date of SpVT diagnosis and the date of death due to any cause or censoring (Page 5, Line 96-97).*

*; Changes in SpVT were assessed by the abdominal imaging studies (CT or MRI) during the follow-up and the imaging examination interval was determined according to the clinical judgment of the attending physicians. (Page 4, Line 80-82)
; Of the 9 patients who were treated with anticoagulation, 2 gastric cancer patients had bleeding events during the anticoagulation therapy. One patient was diagnosed with SpVT (multifocal thrombus in the portal veins) and a pseudoaneurysm in right proximal hepatic artery adjacent to metastatic tumor was also detected at the same time. During dalteparin administration, massive bleeding from the pseudoaneurysm developed and the patient died of bleeding. The other patient was diagnosed with SpVT with disease progression, and bleeding from primary gastric cancer occurred during dalteparin administration. After posterior gastric artery embolization, the patient continued the chemotherapy (Page 10-11, Line 160-168)*

**#2-2)**
- how the diagnosis of Splanchnic vein thrombosis is performed and how patients with splanchnic vein thrombosis is managed (e.g. anticoagulant regimens administered).

***=> Reply #2-2)*** *Thank you for the important comment. As noted in the "Materials and methods" section, diagnosis of SpVT was made by either abdominal computed tomography or magnetic resonance imaging. Reflecting the reviewer’s comment, we have added some sentences about the anticoagulant treatment performed as shown below.*

***Changes in the text #2-2)*** *- Among the 51 patients, 42 (82.4%) did not receive any anticoagulation therapy for SpVT, while 9 (17.6%) received either low molecular weight heparin, warfarin, or direct oral anticoagulant. Four patients (7.8%) received anticoagulation treatment at the time of initial SpVT diagnosis: in 2 patients, SpVT was incidentally detected after surgery and anticoagulant was performed considering the possibility of worsening in the postop period; in the other two cases, SpVT was identified with tumor progression and anticoagulation was conducted for symptom management (in the first patient, ascites accompanying portal vein thrombosis was observed, and in the other case, DVT was detected simultaneously with SpVT). In five other patients (9.8%), anticoagulation therapy was initiated at the time of SpVT aggravation (extension of pre-existing SpVT or development of new SpVT) (Page 10, Line 151-160).*

**#2-3)**
-duration of follow-up and how follow-up is performed (e.g. ultrasonography or computed tomography for vein recanalization or thrombosis progression).

***=> Reply #2-3)*** *We appreciate your suggestion. As noted above (#2-1), changes in SpVT were assessed by the abdominal imaging examination during the follow-up. In addition, we have described the duration of follow-up in the result section (Page 6, Line 114-117).*

**#2-4)**
Furthermore, authors should better explain the statistical methods, how they chose a priori the variables included in the model, and any sensitivity analysis performed.

***=> Reply #2-4)*** *We appreciate your constructive suggestions. Per reviewer’s comment, we have added the additional comments about the statistical methods in the "Materials and methods" section and as shown below.*

***Changes in the text #2-4)*** *- Variables with P-value < 0.05 in the univariable analysis were included in the multivariable analysis (Page 5, Line 101-102).*

*In addition, this study was an observational study tracking the clinical course of cancer patients diagnosed with SpVT. We conducted additional analysis to confirm our observation as below.*

*- We already conducted 2 separate multivariable analyses (Table 3 and Supplementary table 1 (S1 Table)). The ‘enter’ method was used to insert variables into the multivariable analysis model. In both analyses, ECOG PS was a strong prognostic factor. As mentioned in page 13, Line 210, high inter-correlation was observed between tumor stage and clinical situation at the time of diagnosis of SpVT. Therefore, these two variables were not included in the model at the same time, but were included as independent variables in each model separately. As seen in Table 3 and S1 Table, besides ECOG PS, both tumor stage and clinical situation at the time of diagnosis of SpVT were significant prognostic factors in each multivariable analysis. This fact, we believe, indirectly suggests that our multivariable analysis models provide robust results.*

*- Reflecting the reviewer’s suggestion, multivariable analyses were additionally performed using ‘forward’ and ‘backward’ Cox regression analyses when independent variables are included. In both forward and backward Cox regression analyses, both ‘ECOG PS’ and ‘clinical situation at the diagnosis of SpVT’ were the independent prognostic factor for OS, which is exactly same with the results presented in the manuscript (Table 3).*

**Forward Cox regression**

|  | n | Median (months) | 3-year OS rate | P |  | HR | 95% CI | P |
| --- | --- | --- | --- | --- | --- | --- | --- | --- |
| **ECOG performance status** |  |  |  | <0.001 |  |  |  | 0.001 |
| 0 | 15 | 29.1 | 49.2% |  |  | 1.00 | - | - |
| 1 | 30 | NR | 54.0% |  |  | 0.47 | 0.16-1.36 | 0.164 |
| ≥ 2 | 6 | 1.6 | 0.0% |  |  | 10.95 | 1.66-72.23 | 0.013 |
| **Clinical situation at the diagnosis of SpVT** |  |  |  | <0.001 |  |  |  | 0.002 |
| After surgery | 14 | NR | 78.8% |  |  | 1.00 | - | - |
| Initial diagnosis of cancer or tumor recurrence (after curative therapy) | 12 | 18.5 | 22.2% |  |  | 9.10 | 1.84-44.95 | 0.007 |
| During chemotherapy (without tumor progression) | 11 | NR | 87.5% |  |  | 0.81 | 0.07-8.96 | 0.861 |
| During chemotherapy (with tumor progression) | 10 | 5.3 | 15.2% |  |  | 17.37 | 3.05-98.99 | 0.001 |
| Terminal phase (no more chemotherapy) | 4 | 1.2 | 0.0% |  |  | 31.95 | 4.35-234.74 | 0.001 |

**Backward Cox regression**

|  | n | Median (months) | 3-year OS rate | P |  | HR | 95% CI | P |
| --- | --- | --- | --- | --- | --- | --- | --- | --- |
| **Age** |  |  |  | 0.011 |  |  |  |  |
| < 70 years | 41 | NR | 56.6% |  |  | 1.00 | - | - |
| ≥ 70 years | 10 | 13.2 | 0.0% |  |  | 2.09 | 0.67-6.51 | 0.203 |
| **ECOG performance status** |  |  |  | <0.001 |  |  |  | 0.006 |
| 0 | 15 | 29.1 | 49.2% |  |  | 1.00 | - | - |
| 1 | 30 | NR | 54.0% |  |  | 0.51 | 0.17-1.52 | 0.228 |
| ≥ 2 | 6 | 1.6 | 0.0% |  |  | 9.01 | 1.06-76.61 | 0.044 |
| **SpVT-related symptoms** |  |  |  | 0.003 |  |  |  |  |
| Absent | 46 | 29.1 | 50.3% |  |  | 1.00 | - | - |
| Present | 5 | 5.3 | 20.0% |  |  | 1.92 | 0.41-9.03 | 0.411 |
| **Clinical situation at the diagnosis of SpVT** |  |  |  | <0.001 |  |  |  | 0.003 |
| After surgery | 14 | NR | 78.8% |  |  | 1.00 | - | - |
| Initial diagnosis of cancer or tumor recurrence (after curative therapy) | 12 | 18.5 | 22.2% |  |  | 5.57 | 1.00-33.46 | 0.051 |
| During chemotherapy (without tumor progression) | 11 | NR | 87.5% |  |  | 0.80 | 0.72-8.91 | 0.857 |
| During chemotherapy (with tumor progression) | 10 | 5.3 | 15.2% |  |  | 14.07 | 2.36-83.82 | 0.004 |
| Terminal phase (no more chemotherapy) | 4 | 1.2 | 0.0% |  |  | 33.32 | 4.41-251.93 | 0.001 |

**#2-5)** Results: authors should consider to repeat survival analysis with a short time of follow-up (e.g., 6 or 12 months). Patients with cancer and splanchnic vein thrombosis generally have a short prognosis and after the first year of follow-up mortality may be affected by other variables.

***=> Reply #2-5)*** *Thank you for the important comment. Per the reviewer’s comment, we have repeated survival analysis with a shorter time of follow-up (12 months) and added the additional descriptions to the revised manuscript as shown below. The cut off at 6 months was not appropriate because there were too few death events.*

***Changes in the text #2-5)***  *- Survival analysis with a short time of follow-up (see S2 Table). In this multivariable analysis, ECOG PS, clinical situation at the time of diagnosis of SpVT and serum albumin level at the time of initial SpVT diagnosis were independently significant prognostic factors.*

*- The following sentences were added to the revised manuscript.
; Moreover, considering the period when SpVT can have a significant impact on survival, another survival analysis was repeated with a shorter follow-up period (12 months). In univariable analyses (S2 Table), ECOG PS, tumor stage, SpVT-related symptoms, clinical situation at the time of diagnosis of SpVT, albumin level, and hemoglobin level were associated with OS after the diagnosis of SpVT (P < 0.05). In multivariable analysis (S2 Table), ECOG PS, clinical situation at the time of diagnosis of SpVT, and albumin level were independently significant prognostic factors. (Page 14, Line 219-225)
; ECOG PS and clinical situation at the time of diagnosis of SpVT were independent prognostic factors in both short-term and long-term survival analyses. In short-term survival analysis, albumin level was also an independent prognostic factor, which is consistent with the results of previous studies in cancer patients [14, 15]. (Page 17, Line 289-292)*

**#2-6)** Discussion: please report in first paragraph the major findings of the study.
Please consider to shorten the Discussion as some parts appear confusing and repetitive.

***=> Reply #2-6)*** *Thank you for the important comment. Reflecting the reviewer’s comment, we have reported the major findings in the first paragraph as shown below and shortened the discussion section.*

**Changes in the text #2-6)**
*- SpVT occurred in various clinical situations and most of them did not cause symptoms or had little impact on the patient’s cancer treatment course. During the follow-up period, in our patient cohort, there were no cases of death due to SpVT, and all deaths were caused by tumor progression. In the multivariable survival analysis, ECOG PS and clinical situation at the time of SpVT diagnosis were significant prognostic factors. (Page 14, Line 229-233)*

*- The following sentences have been deleted from the previous manuscript*

*; (in Page 14) ~~Risk factors influencing survival in these patients with SpVT were also investigated.~~*

*; (in Page 17) ~~Most cases of SpVT are incidentally diagnosed and do not cause symptoms. In our patient cohort, only 7.8% of the patients (4/51) received anticoagulation at the initial diagnosis of SpVT. Among patients who had not received anticoagulation at the time of SpVT diagnosis, five out of 47 patients (10.6%) underwent anticoagulation due to aggravation of SpVT.~~*

*; (in Page 17) ~~As mentioned above, most guidelines suggest that the treatment of incidentally found SpVT should be determined on a case-by-case basis, considering the potential benefits and risks of anticoagulation.~~*

**Minor comments:**

**#2-7)** Please report the number of patients lost to follow-up in the first part of Results section

***=> Reply #2-7)*** *Following the reviewer’s comment, we have added an additional sentence as shown below.*

***Changes in the text #2-6)***  *- One patient immigrated to a foreign country (follow-up duration; 6.6 months), so further survival follow-up could not be done. (Page 6, Line 117-118)*

**#2-8)** Please be consistent with the use of "gastric cancer" in the text and tables

***=> Reply #2-8)*** *Thank you for the suggestion. Following the reviewer’s comment, we unified the terminology as ‘gastric cancer’.*

**#2-9)** Please replace "asymptomatic" or "incidental" thrombosis with "incidentally-detected thrombosis"

***=> Reply #2-9)*** *Thank you for the suggestion. Following the reviewer’s comment, we replaced the "asymptomatic" or "incidental" thrombosis with "incidentally detected” thrombosis.*

**#2-10)** on row 114 page 7, please specify the type of other thrombosis

***=> Reply #2-10)*** *Thank you for the comment. As noted in the Table 2, the other sites were gastric vein, right hepatic vein, and internal iliac vein. As per reviewer’s comment, we specified the locations as shown below.*

***Changes in the text #2-10)***  *- The anatomical sites of SpVT development were the portal vein (66.7%), superior mesenteric vein (15.7%), inferior mesenteric vein (3.9%), others (5.9%; gastric vein (2.0%), right hepatic vein (2.0%), and internal iliac vein (2.0%)), and multiple sites (7.8%). (Page 8, Line 134)*
