# Supplementary material for: Clinical characteristics and disease course of splanchnic vein thrombosis in gastrointestinal cancers: A prospective cohort study
Source: PLoS One. 2022 Jan 18;17(1):e0261671. doi: 10.1371/journal.pone.0261671 (PMC8765650; doi:10.1371/journal.pone.0261671)
Supplement: Attachment — Submitted filename: Response to Reviewers.docx [file pone.0261671.s008.docx]

-October, 2021

**Editor-in-Chief: Dr. Emily Chenette; *PLOS ONE***

**Dear Dr. Chenette,**

Thank you very much for your insightful review of our manuscript. All the comments were very helpful to improve the quality of this report, and we did our best to address your detailed comments and suggestions. Adhering to the respectful recommendations suggested by the Editor and the reviewers, we have revised our manuscript. Point-by-point revisions are outlined in detail below.

All of the authors have read and agreed with the revised manuscript. Again, thank you very much for considering our work for publication in your journal. This research was partially funded by the Seoul National University Bundang Hospital Research Fund (Number: 02-2017-036). There was no additional external funding received for this study.

Along with my coauthors, I would like to ask you to consider the attached manuscript for publication in ***'PLOS ONE***' as an original article.

Best regards,

Keun-Wook Lee, M.D., Ph.D.

Professor, Department of Internal Medicine, Seoul National University Bundang Hospital, Seoul National University College of Medicine

82 Gumi-ro 173 Beon-gil, Bundang-gu, Seongnam 13620, Republic of Korea

Phone: +82-31-787-7039; E-mail: hmodoctor@snu.ac.kr

**PONE-D-21-10010R1**

Clinical characteristics and disease course of splanchnic vein thrombosis in gastrointestinal cancers: A prospective cohort study

PLOS ONE

**Academic editor:** I understand that a power calculation was not performed beforehand. Please perform one post-hoc?

***=> Reply)*** *Thank you for the important comment. Because it was a prospective observational study, we did not establish a hypothesis or perform a power calculation in the paper. However, in this page, we summarized the results of post hoc analysis by setting the hypothesis as follows.*

*Null Hypothesis (H0): P0 of SpVT patients will need anticoagulant therapy.*

*Alternative hypothesis (H1): P1 of SpVT patients will need anticoagulant therapy.*

*Our study: 9 out of 51patients (9/51, 0.176=P1) received anticoagulant treatment.*

***Numeric Results for Testing One Proportion using the Z-Test with S(P0) and C.C. ─────────────────***

*Alternative Hypothesis:	Two-Sided	(H0: P = P0 vs. H1: P ≠ P0)*

***Proportion	Proportion***

***Given H0	Given H1	Difference	Reject H0***

***Power*	n	P0	P1	P1-P0	Alpha	If |Z| >***

*0.15282	51	0.25	0.176	-0.074	0.05	1.96*

*0.41410	51	0.30	0.176	-0.124	0.05	1.96*

*0.73379	51	0.35	0.176	-0.174	0.05	1.96*

*0.93260	51	0.40	0.176	-0.224	0.05	1.96*

*0.99167	51	0.45	0.176	-0.274	0.05	1.96*

*0.99955	51	0.50	0.176	-0.324	0.05	1.96*

** Power was computed using the normal approximation method.*

***References***

*Chow, S. C., Shao, J., and Wang, H. 2008. Sample Size Calculations in Clinical Research, Second Edition.*

*Chapman & Hall/CRC. Boca Raton, Florida.*

*Fleiss, J. L., Levin, B., and Paik, M.C. 2003. Statistical Methods for Rates and Proportions. Third Edition. John*

*Wiley & Sons. New York.*

*Lachin, John M. 2000. Biostatistical Methods. John Wiley & Sons. New York.*

*Machin, D., Campbell, M., Fayers, P., and Pinol, A. 1997. Sample Size Tables for Clinical Studies, 2nd Edition.*

*Blackwell Science. Malden, Mass.*

*Ryan, Thomas P. 2013. Sample Size Determination and Power. John Wiley & Sons. Hoboken, New Jersey.*

*Zar, Jerrold H. 2010. Biostatistical Analysis (Fifth Edition). Prentice-Hall. Englewood Cliffs, New Jersey.*

***Summary Statements ─────────────────────────────────────────────────────────***

*(1) A sample size of 51 achieves 73.379% power to detect a difference (P1-P0) of -0.174 using a two-sided Z-test that uses S(P0) to estimate the standard deviation and has a continuity correction with a significance level (alpha) of 0.05. These results assume that the population proportion under the null hypothesis (P0) is 0.35.*

*(2) A sample size of 51 achieves 99.955% power to detect a difference (P1-P0) of -0.324 using a two-sided Z-test that uses S(P0) to estimate the standard deviation and has a continuity correction with a significance level (alpha) of 0.05. These results assume that the population proportion under the null hypothesis (P0) is 0.50.*

***Chart Section ──────────────────────────────────────────────────────────────***

***As mentioned earlier, this analysis was not reflected in the paper as it was an unscheduled post hoc analysis.***

**Reviewer #1**:

The study makes a small yet important contribution to the field of GI malignancies related splanchnic vein thrombosis, which has so far been under-researched. It is presented in an easily comprehensible manner with sound statistical analysis and comprehensive data collection of important variables in the study cohort.

*=> Thank you for the constructive and valuable comments.*

**Reviewer #2**:

*=> Thank you for the constructive and valuable comments and suggestions. We revised our manuscript following your suggestions. Please refer to the manuscript file with track changes.*

**Major comments:**

In the "Materials and methods" section:

**#2-1)**
Please evaluate to better specify the outcomes evaluated. In Results both recurrent event and bleeding were reported without any clarification in the Methods. Furthermore, authors should describe as outcome were defined and evaluated.

***=> Reply #2-1)*** *We appreciate your constructive suggestion. Reflecting the reviewer’s comments, we have revised the manuscript as shown below. After revision, all the variables analyzed in the result are mentioned in the "Materials and methods" section.*

***Changes in the text #2-1)*** *- The following data were collected for this study: (1) demographics such as age, sex, height, weight, comorbid disease, and Eastern Cooperative Oncology Group performance status (ECOG PS); (2) data related to gastrointestinal cancer such as diagnosis code, date of diagnosis, laboratory blood test results, abdominal CT or MRI, surgery, chemotherapy, and radiation therapy; (3) data related to SpVT such as location, date of identification, initial symptoms and signs (abdominal pain, hepatomegaly, ascites, etc.), treatment, change of SpVT-related symptoms, recurrent thrombosis (including extension of pre-existing SpVT or new SpVT) during follow-up, and bleeding events after anticoagulation therapy; and (4) survival data, date of death, and reason for death in cases of death. (Page 4-5, Line 85-92).*

*- The outcomes of interest in this observational study were clinical characteristics and course of SpVT including anticoagulation therapy, recurrent thrombosis and bleeding events during follow-up, and risk factors influencing survival in patients with SpVT. Data were analyzed using IBM SPSS Statistics (IBM Corp., Armonk, NY, USA). The Kaplan–Meier method was used to calculate the overall survival (OS). OS was defined as the time period between the date of SpVT diagnosis and the date of death due to any cause or censoring. In univariable analyses, log-rank tests were performed to examine differences in survival outcomes among the comparison groups. Age, sex, tumor-related characteristics (primary tumor, pathology and stage), parameters related to the patient's status at the time of diagnosis of SpVT (ECOG PS and laboratory blood test results (hemoglobin level, white blood cell count, platelet count and albumin level), and parameters related to SpVT (location, the presence of related symptoms and the clinical situation at the time of SpVT diagnosis) were included in the univariable analyses. (Page 5, Line 94-106).*

**#2-2)**
Authors should clearer report statistical methods used and the sensitivity analysis performed. Furthermore, they should report in methods the univariable and multivariable models actually reported in the Results. Furthermore, they should report a-priori the variable they evaluate in the model.

***=> Reply #2-2)*** *Thank you for the important comment. Reflecting the reviewer’s comments, we have revised the "Materials and methods" section as shown below. In addition, in previous revision process, multivariable analyses were additionally performed using ‘forward’ and ‘backward’ Cox regression analyses. In both forward and backward Cox regression analyses, both ‘ECOG PS’ and ‘clinical situation at the diagnosis of SpVT’ were the independent prognostic factor for OS, which is exactly same with the results presented in the manuscript (Table 3, 'enter' method).*

***Changes in the text #2-2)*** *- Cox proportional hazards models were used to analyze the influence of specified risk factors on survival outcomes in the multivariable analysis. Variables with P-value < 0.05 in the univariable analysis were included using the ‘enter’ method in the multivariable Cox proportional hazards model analysis. If there was high intercorrelation between the selected variables, one variable was selected and included in the multivariable analysis model, and it was checked whether the analysis result was the same when the other variable was selected instead of the first selected one. P-values less than 0.05 were considered significant. (Page 5-6, Line 106-112).*

In the "Results" section:

**#2-3)**
- to report time from surgery, chemotherapy or radiotherapy from thrombosis development (e.g., < or > 3 months), if available

***=> Reply #2-3)*** *Thank you for the important comment. Reflecting the reviewer's comment, we have modified the Table 1 as shown below. (Page 7, Table 1).*

| Major surgery |  |  |  |  |
| --- | --- | --- | --- | --- |
| No | 10 (19.6%) | 7 (28.0%) | 3 (12.5%) | - |
| Yes (>3 months) | 27 (52.9%) | 14 (56.0%) | 13 (54.2%) | - |
| Yes (≤3 months^e^) | 14 (27.5%) | 4 (16.0%) | 8 (33.3%) | 2 (100.0%) |
| Radiotherapy |  |  |  |  |
| No | 39 (76.5%) | 20 (80.0%) | 18 (75.0%) | 1 (50.0%) |
| Yes (>3 months) | 10 (19.6%) | 5 (20.0%) | 4 (16.7%) | 1 (50.0%) |
| Yes (≤3 months) | 2 (3.9%) | 0 (0.0%) | 2 (8.3%) | - |
| Chemotherapy |  |  |  |  |
| No | 13 (25.5%) | 6 (24.0%) | 7 (29.2%) | - |
| Yes (>3 months) | 6 (11.8%) | 1 (4.0%) | 4 (16.7%) | 1 (50.0%) |
| Yes (≤3 months) | 32 (62.7%) | 18 (72.0%) | 13 (54.2%) | 1 (50.0%) |
| ^e^Within 3 months prior to diagnosis of SpVT. | | | | |

**#2-4)**
- to report in the text the number of patient with different type of thrombosis or with different rate of recanalization along with proportions. This may increase the readability of results.

***=> Reply #2-4)*** *We appreciate your constructive suggestions. Per reviewer’s comment, we have added the additional comments in Table 2 and we have revised the text of manuscript as shown below.*

***Changes in the text #2-4)*** *- ^j^Rates of recanalization according to location of SpVT were as follows: portal vein 47.1% (16/34); superior mesenteric vein 50.0% (4/8); inferior mesenteric vein 50.0% (1/2); others 33.3% (1/3); and multiple sites 50.0% (2/4). (Page 9, Table 2).*

*- The clinical course of SpVT during the follow-up varied: (1) spontaneous resorption without any anticoagulation in 24 patients (47.1%); (2) resorption with anticoagulation in 2 (3.9%); (3) persistent thrombosis without progression in 9 (17.6%); (4) extension of SpVT within the same vein in 12 (23.5%); and (5) extension to other adjacent veins beyond the existing location or new SpVT occurrence in 4 (7.8%). Rates of spontaneous recanalization according to location of SpVT development were as follows: portal vein 47.1% (16/34); superior mesenteric vein 50.0% (4/8); inferior mesenteric vein 50.0% (1/2); others 33.3% (1/3); and multiple sites 50.0% (2/4). More detailed information on the clinical course of SpVT during the follow-up is shown in Table 2. (Page 11, Line 175-183).*

**#2-5**
- to report more detailed information on anticoagulant therapy (e.g. number of patients treated with different type of anticoagulant therapy, duration of anticoagulation).

***=> Reply #2-5)*** *Thank you for the important comment. Per the reviewer’s comment, we added the additional descriptions to the revised manuscript as shown below.*

***Changes in the text #2-5)***  *- Among the 51 patients, 42 (82.4%) did not receive any anticoagulation therapy for SpVT, while 9 (17.6%) received either low molecular weight heparin (n=6), warfarin (n=1), or direct oral anticoagulant (n=2). In the 9 patients who were treated with anticoagulation, the median treatment duration was 4.7 months (range, 0.2–6.4 months). (Page 10, Line 153-156).*

**#2-6**
- to report the number of outcomes events in both anticoagulated patients and those that remained untreated.

***=> Reply #2-6)*** *Thank you for the important comment. Per the reviewer’s comment, we added the additional descriptions to the revised manuscript as shown below.*

*- Rates of recanalization of SpVT were 57.1% (24/42) in non-anticoagulation group and 22.2% (2/9) in anticoagulation group. (Page 10, Line 156-158).*

In the " Discussion and conclusions" section:

**#2-7)**
Discussion may be shortened to increase readability.

***=> Reply #2-7)*** *Thank you for the constructive suggestion. Reflecting the reviewer’s comments, we have the revised manuscript as shown below.*

**Changes in the text #2-7)**

*- ~~Our study has shown that, in patients with gastrointestinal cancer, incidental SpVT is more common than symptomatic SpVT.~~ In our patient cohort, most incidentally detected acute or chronic SpVT did not require anticoagulation. (Page 18, Line 300).*

*- ~~In patients with active cancer that cannot be removed, the use of anticoagulants should be decided more prudently because long-term, probably indefinite, anticoagulation would be necessary once anticoagulation is started.~~ (Page19, Line 319).*

*- ~~Future researches are needed to establish the best anticoagulation method for the treatment of SpVT in patients with cancer.~~ (Page 21, Line 321).*

**#2-8)**
Conclusion should better report the results of the study (e.g. reported data do not permit to draft any conclusion on anticoagulant therapy).

***=> Reply #2-8)*** *Thank you for the important comment. I think the content of the paper was refined during the revision process above.* *The key point is that in most gastrointestinal cancer patients, SpVT does not affect survival, thus it can be monitored without anticoagulation intervention. Reflecting the reviewer’s comments, we have the revised manuscript as shown below.*

**Changes in the text #2-8)**

*- In patients with gastrointestinal cancer, most SpVT cases are asymptomatic and incidentally found. Most cases of SpVT had little effect on the patient’s cancer treatment journey. Therefore, except for symptomatic SpVT cases, we suggest that clinicians could adopt a strategy of close observation and symptom monitoring regardless of whether SpVT is acute or not, even in patients with active cancer. Anticoagulation should be considered only for SpVT cases selected strictly, weighing the risks and benefits. (Page 20, Line 335-340).*

**Minor comments:**

**#2-9)** Author should correct typo and grammatical errors.

***=> Reply #2-9)*** *Thank you for the important comment. Once again, the manuscript has been carefully reviewed and revised to the best of our ability.*

**#2-10)** Author should consider include in introduction and discussion recent data from prospective studies on clinical course of cancer patients with splanchnic vein thrombosis.

***=> Reply #2-10)*** *Thank you for the suggestion. Most other studies on SpVT have been cited in our paper. One research was published officially during the revision process, and the information has been updated as follows.*

17.	Valeriani E, Di Nisio M, Riva N, Cohen O, Garcia-Pagan JC, Magaz M, et al. Anticoagulant therapy for splanchnic vein thrombosis: a systematic review and meta-analysis. Blood. 2021;137(9):1233-40. Epub 2020/09/11. doi: 10.1182/blood.2020006827. PubMed PMID: 32911539.
