# Supplementary material for: Clinical characteristics and disease course of splanchnic vein thrombosis in gastrointestinal cancers: A prospective cohort study
Source: PLoS One. 2022 Jan 18;17(1):e0261671. doi: 10.1371/journal.pone.0261671 (PMC8765650; doi:10.1371/journal.pone.0261671)
Supplement: Attachment — Submitted filename: Response to Reviewers.docx [file pone.0261671.s009.docx]

- December, 2021

**Editor-in-Chief: Dr. Emily Chenette**

**Academic Editor: Pal Bela Szecsi**

**Dear Dr. Chenette & Szecsi,**

Thank you very much for insightful review of our manuscript. The power calculation you strongly suggested has been added in Discussion and also with an appendix.

All of the authors have read and agreed with the revised manuscript. Again, thank you very much for considering our work for publication in your journal. This research was partially funded by the Seoul National University Bundang Hospital Research Fund (Number: 02-2017-036). There was no additional external funding received for this study.

Along with my coauthors, I would like to ask you to consider the attached manuscript for publication in ***'PLOS ONE***' as an original article. I appreciate your constructive suggestion again.

Best regards,

Keun-Wook Lee, M.D., Ph.D.

Professor, Department of Internal Medicine, Seoul National University Bundang Hospital, Seoul National University College of Medicine

82 Gumi-ro 173 Beon-gil, Bundang-gu, Seongnam 13620, Republic of Korea

Phone: +82-31-787-7039; E-mail: hmodoctor@snu.ac.kr

**PONE-D-21-10010R2**

Clinical characteristics and disease course of splanchnic vein thrombosis in gastrointestinal cancers: A prospective cohort study

PLOS ONE

**Academic editor:** Both reviewers are satisfied, however I request that you include a paragraph regarding the power calculation you performed. It strength the statement of use of anticoagulation, especially considering the relative few patients in the study.

***=> Reply)*** *Thank you for the important comment. We have added the power calculation as an appendix and mentioned it in the manuscript as shown below.*

***Changes in the text)***

*We conducted post hoc power analysis. In this study, 9 out of 51 patients (9/51 = 0.176 [P1]) received anticoagulant treatment. When it was assumed that about one-third of patients with SpVT will require anticoagulation (P0 = 0.35 [null hypothesis]), the sample size of 51 achieved 73.379% power to detect a difference (P1-P0) of -0.174 with a significance level (alpha) of 0.05. When it was assumed that about half of patients with SpVT will require anticoagulation (P0 = 0.50), the sample size of 51 achieved 99.955% power to detect a difference (P1-P0) of -0.324 (alpha = 0.05; see S1 Appendix for post hoc power analysis). (Page 19, Line 327-333)*
